# A Weapon Against Implant-Associated Infections: Antibacterial and Antibiofilm Potential of Biomaterials with Titanium Nitride and Titanium Nitride-Silver Nanoparticle Electrophoretic Deposition Coatings

**DOI:** 10.3390/ijms26041646

**Published:** 2025-02-14

**Authors:** Sandra Hojda, Maria Biegun-Żurowska, Alicja Skórkowska, Karolina Klesiewicz, Magdalena Ziąbka

**Affiliations:** 1 Department of Pharmaceutical Microbiology, Faculty of Pharmacy, Jagiellonian University Medical College, 30-688 Kraków, Poland; sandra.hojda@uj.edu.pl; 2 Department of Ceramics and Refractories, Faculty of Materials Science and Ceramics, AGH University of Krakow, 30-059 Kraków, Poland; biegun@agh.edu.pl; 3 BioImaging Laboratory, Center for the Development of Therapies for Civilization and Age-Related Diseases, Jagiellonian University Medical College, 30-688 Kraków, Poland; alicja.skorkowska@uj.edu.pl

**Keywords:** titanium nitride (TiN), silver nanoparticles (AgNPs), antibacterial activity, antibiofilm properties, biomaterials, medical implants, biofilm formation, *Escherichia coli*, *Staphylococcus aureus*, *Enterococcus faecium*, *Enterococcus faecalis*

## Abstract

Implant-associated infections are a frequent complication of surgeries involving biomaterial implants. *Staphylococcus* and *Enterococcus* species are the leading causes of infections linked to bone-anchored and joint implants. To address this challenge, developing antibacterial coatings to prevent bacterial attachment and biofilm formation on biomaterials is critical. This study aimed to evaluate the antibacterial and antibiofilm properties of two biomaterial coatings: titanium nitride (TiN) and titanium nitride with silver nanoparticles (TiN/Ag). Antibacterial activity was tested against common biofilm-forming pathogens, including *Escherichia coli*, *Staphylococcus aureus*, *Enterococcus faecalis*, and *Enterococcus faecium*. The results demonstrated that both coatings significantly reduced bacterial cell counts, with the TiN/Ag coating showing superior performance due to the addition of silver nanoparticles. This enhancement was particularly effective in reducing biofilm formation across all the tested strains, with the most pronounced effects observed for *E. faecium* and *E. faecalis*. The silver nanoparticles synergistically improved the antibiofilm properties of the TiN coating, efficiently disrupting biofilm integrity and reducing bacterial adhesion. By reducing bacterial attachment and biofilm formation on biomaterial surfaces, TiN/Ag coatings offer a promising strategy to minimize complications associated with biomaterial implants. These findings highlight the potential of TiN and TiN/Ag coatings for medical applications.

## 1. Introduction

Implant-associated infections are a common problem regarding biomaterials used in medical implants, particularly for hard tissues, such as bones or teeth. Among other causes, they may stem from a bacterial biofilm forming on the surface of medical devices. Even the smallest infection can lead to serious complications, making biofilm formation a significant clinical challenge for researchers. As such infections are highly resistant to standard antibiotic therapies and immune response, they may cause a deterioration of patients’ health [1,2].

Biofilms are called biological membranes which are a group of bacteria cells adhering to surfaces surrounded by extracellular matrix. Such a structure provides protection to the bacteria from external threats (including antibiotics or environmental factors) and thus bacteria stay longer on the surface. This phenomenon is extremely dangerous in the case of medical devices, possibly leading to many complications. The increased morbidity and mortality make the biofilm a critical problem in modern medicine. There are many strains capable of forming biofilm in the medical environment but the most common pathogens with that ability are *Staphylococcus aureus* (*S. aureus*), *Enterococcus faecalis* (*E. faecalis*), and *Enterococcus faecium* (*E. faecium*). These bacteria can colonize biomaterials, leading to persistent infections [3,4]. A common reason for implant removal is the difficulty in eradicating the biofilm once it has been established, due to the heightened resistance of bacteria within these structures. The resistance extends to antibodies, phagocytic immune cells, and conventional antibacterial therapies. Therefore, it is crucial to explore new antibacterial coatings that can prevent bacterial attachment and biofilm formation on biomaterials [5].

Biomaterials aimed to replace hard tissues have to meet strict requirements in terms of cytotoxicity or mutagenicity, as well as biocompatibility, mechanical strength, and resistance to bacteria colonization [6]. Biomaterials like titanium and its alloys are commonly used in medical applications due to their beneficial characteristics. Various coatings have been explored for their potential to reduce bacterial attachment and enhance antimicrobial properties [4,7]. Studies have shown that titanium nitride silver coating materials can enhance antibacterial effects. Nevertheless, these materials face challenges related to microbial infections [8].

The Ti-6Al-4V titanium alloy is often used in medicine. It is characterized by high strength, fracture toughness, and corrosion resistance which are desired properties. Still, it can cause undesirable reactions after the implementation. There is always a risk of issues generated by infections, ions release, or collagen formation. Ongoing research focuses on alleviating implantation-related risks. Titanium-based materials, also including titanium nitride (TiN) and its composites, are commonly used in implants, especially orthopedic and dental ones, due to their excellent mechanical properties, corrosion resistance, and biocompatibility. Moreover, TiN is known for its low toxicity and is regarded as a probable antibacterial agent. One of the promising solutions is the use of silver nanoparticles (AgNPs) in titanium nitride coatings. Their incorporation enhances antibacterial properties, as silver is widely recognized for strong antimicrobial activity, including the one against antibiotic-resistant strains. Recent studies confirm that titanium nitride coatings containing silver nanoparticles reduce bacterial adhesion and biofilm formation on medical implants. These results apply to a wide range of pathogens including the most common ones, e.g., *S. aureus* and *Enterococcus* spp. [9,10,11]. Such a dual approach of TiN/Ag coatings offers several advantages over standard antibacterial coatings, i.e., the ones based solely on silver or polymeric materials. TiN/Ag coatings combine the long-lasting stability and mechanical strength of TiN with the potent antimicrobial effects of silver, providing enhanced protection against bacterial adhesion and biofilm formation [12].

Nowadays, in order to improve medical outcomes, there is a need to develop biomaterials for implants with antibacterial and antibiofilm effects. The materials should be suitable for tissue integration and capable of providing satisfactory protection against biofilm formation. Various strategies are used to address the bacterial biofilm formation on biomaterials. Recent studies have focused on alternative coating technologies to enhance antibacterial properties. For example, hydrophobic nanocomposite coatings were developed to inhibit bacterial adhesion, demonstrating effectiveness against *Staphylococcus aureus* and *Pseudomonas aeruginosa* [13]. Another promising approach involves polymer-based nano-gel coatings which significantly reduce bacterial colonization on polymeric surfaces [14]. Moreover, electroactive coatings, e.g., incorporating poly(3,4-ethylenedioxythiophene) (PEDOT), were investigated for their ability to modulate biofilm formation through external polarization, which offers a smart antibacterial strategy for medical implants [15]. Among these solutions, titanium nitride coatings combined with silver nanoparticles are equally promising in implant-associated infections [10,16] and another standout candidate is chitosan as it combines antibacterial activity with a chemical composition closely resembling the extracellular matrix of bone tissue.

Studies also focus on the abilities of biofilm formation of *S. aureus*, *E. faecalis*, and *E. faecium*—the most common strains causing medical complications regarding implants. After forming biofilm, they are hard to treat with standard antibiotics. Our study aimed to contribute to progress in identifying coatings that can improve implants’ lifespan and reduce the biofilm formation abilities of these strains. Such a solution might reduce the incidence of bacterial infections, which is crucial in case of rising antibiotic resistance [11]. The primary purpose of our study was to thoroughly evaluate the effectiveness of two distinct coatings applied to biomaterials against bacterial strains commonly associated with medical implant infections. The coatings were titanium nitride (TiN/Ch) with chitosan, titanium nitride with chitosan, and silver nanoparticles (TiN/Ch/AgNPs). The novel approach employed a method of electrophoretic deposition (EPD). Unlike other coating techniques, it ensured high controllability and repeatability of the process [9,17]. Previous studies investigated the biological and antibacterial properties of chitosan coatings containing AgNPs and CuNPs, deposited on Ti13Zr13Nb titanium alloy via electrophoresis. The coatings were characterized by good biocompatibility and efficacy against *Staphylococcus aureus* and *Escherichia coli* bacteria. However, these studies did not consider the use of titanium nitride (TiN) as a coating component, which could additionally improve the mechanical and antibacterial properties of the material [18].

Moreover, our coatings were meant not only to limit bacterial adhesion but also exhibit strong antibiofilm properties, which would distinguish them from the previously studied materials. The combination of TiN and AgNPs with chitosan is a novel approach in the field of biomaterials engineering, and the obtained results may be of key importance in the clinical context—especially in preventing infections associated with medical implants [18].

## 2. Results

### 2.1. Effective Deposition and Homogeneity of Antibacterial Coatings

**Figure 1:**
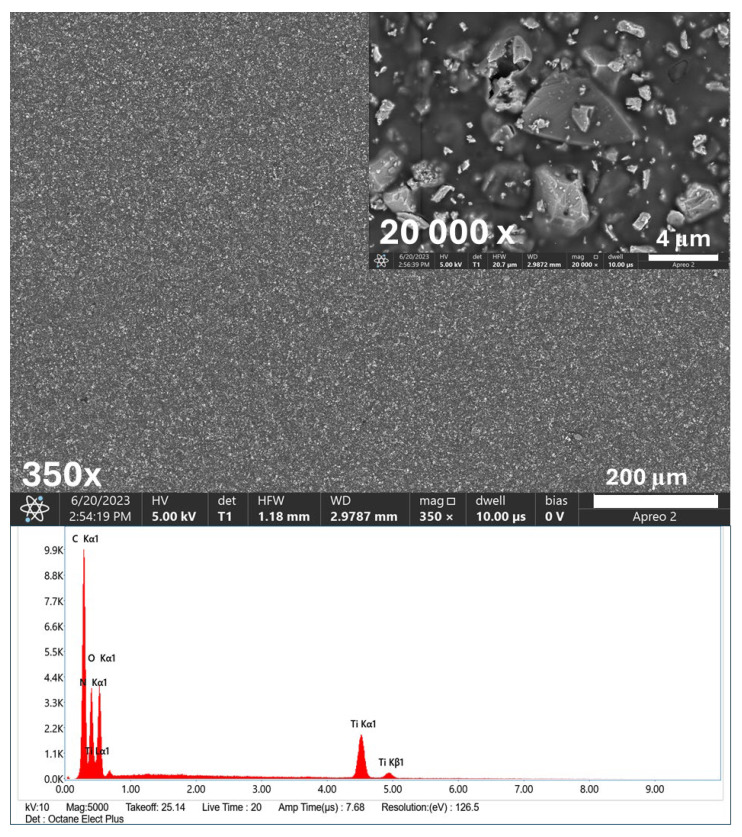
SEM images and EDS spectra with element content of TiAlV/TiN/Chitosan coating.

**Figure 2:**
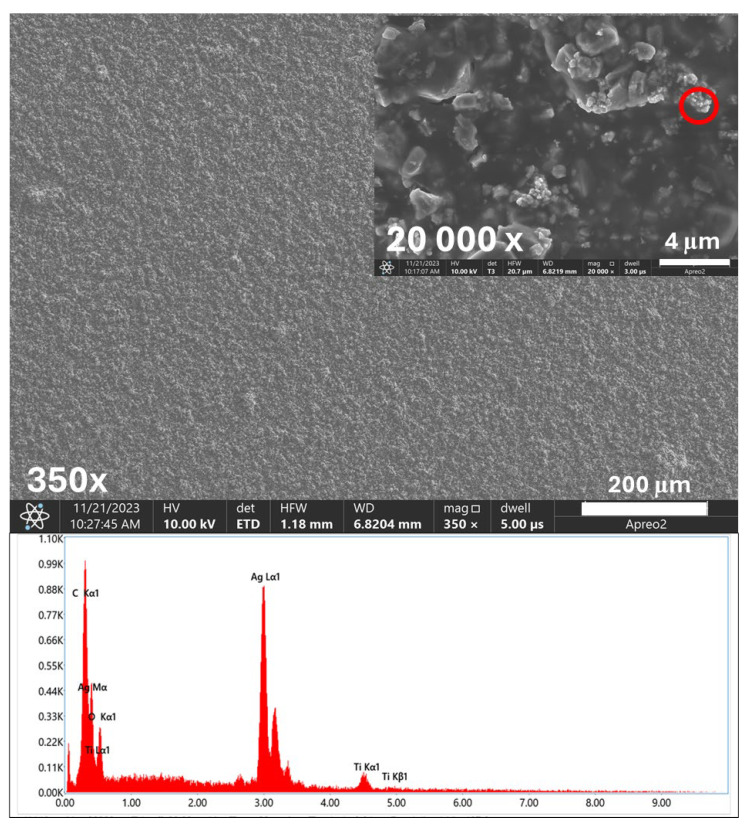
SEM images and EDS spectra with element content of TiAlV/TiN/Chitosan/Ag coating. Red circle indicates occurrence of silver nanoparticles by EDS point analysis.

The SEM images and EDS spectra, along with the elemental composition of the biomaterials studied, are presented in Figure 1 and Figure 2.

The SEM analysis confirmed the successful deposition of titanium nitride (TiN) particles (Figure 1-spectrum) and silver nanoparticles (AgNPs) on the etched Ti6Al4V substrate (Figure 2-spectrum). These observations proved the uniformity and quality of the coating process. The obtained coatings were characterized by a crack-free, homogeneous microstructure, ensuring an even and dense distribution of the deposited TiN particles on the substrate surface. At magnification of 20 Kx, TiN particles measuring more than 2 μm were visible. Figure 2 also shows the distribution of silver nanoparticles that tightly adhered to the larger TiN particles.

The EDS spectra provided a detailed elemental analysis, confirming the presence of titanium (Ti), aluminum (Al), vanadium (V), nitrogen (N), silver (Ag), and carbon (C) from the chitosan layer. Ag nanoparticles are particularly important due to their well-documented antibacterial and antibiofilm properties. In addition, the homogenous coating of the material ensured its optimal contact with the surrounding environment, which is crucial for antibacterial efficacy. The combination of TiN and AgNPs in the chitosan layer created a multifunctional coating with enhanced mechanical stability and antimicrobial activity.

### 2.2. Antibacterial Potential of Tested Coatings

**Figure 3:**
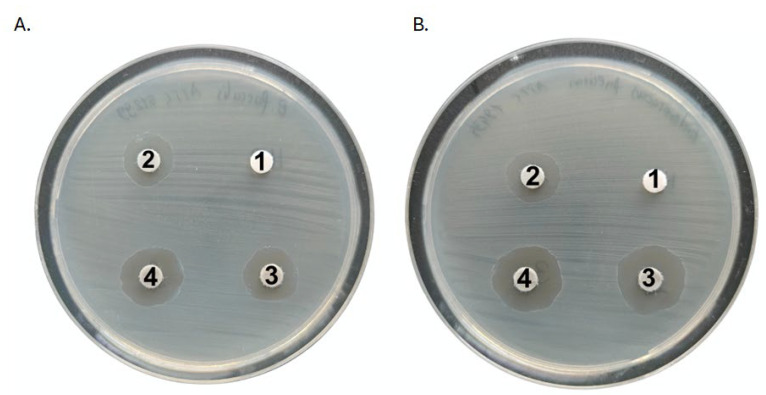
Activity of tested solutions by disk-diffusion method with TiAlV (2) [Φ = 14 mm], TiN/Chitosan (3) [Φ = 14 mm], and TiN/Chitosan/Ag (4) [Φ = 16 mm] against *E. faecalis* ATCC 51299 (**A**) and *E. faecium* ATCC 19434 (**B**) with water as control (1). For *E. faecium* ATCC 19434 (**B**), inhibition zone diameters were as follows: (2) Φ = 13 mm; (3) Φ = 15 mm; (4) Φ = 16 mm.

Firstly, we employed the disk-diffusion method to evaluate the antibacterial activity of the solution containing TiN/Chitosan and TiN/Chitosan/Ag. The antibacterial effects against all the tested bacterial strains were observed for both types of coatings (Table 1, Figure 3). Against *E. coli*, the inhibition zone was bigger for the TiN/Chitosan/Ag in comparison to the TiN/Chitosan. This zone was 12 mm for the TiN/Chitosan coating and increased to 18 mm when silver nanoparticles were added to the coating. In the case of *S. aureus*, the inhibition zone rose from 14 mm (TiN/Chitosan) to 20 mm for the silver-doped coating. Similar effects were observed for *Enterococci* strains. Five strains were tested (two *E. faecalis*, two *E. faecium*, and one *E. gallinarum*) and in each case, the results showed that the addition of chitosan and silver nanoparticles enhanced the antibacterial result. The inhibition zones for these strains’ values ranged between 15 mm and 22 mm. The control disks, which did not have coatings, showed significantly smaller or even no inhibition zones. This proved the effectiveness of the tested coating.

The cutoff was established at 10 mm, based on evaluating of the solvent’s impact on the inhibition zone. The inhibition zone reached 9 mm for *E. coli* and 10 mm for *S. aureus* and *E. faecium* strains.

### 2.3. Reduction in Bacterial Cells on Biomaterials

In the quantitative analysis of the coatings composed of TiAlV/TiN/Chitosan and TiAlV/TiN/Chitosan/Ag, a marked decrease was noticed in bacterial counts for both *S. aureus* and *E. coli*.

**Figure 4:**
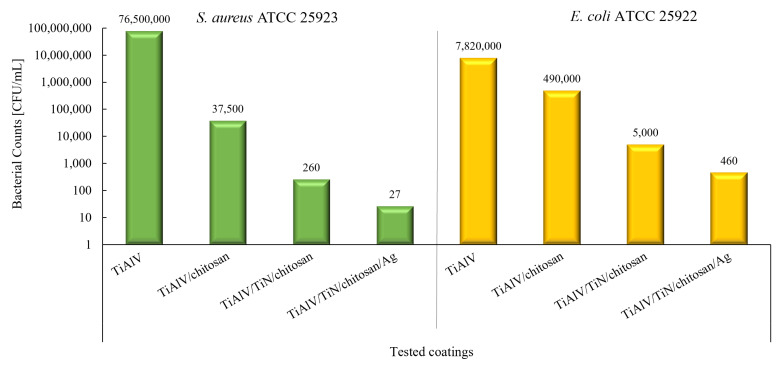
Reduction in bacterial counts [CFU/mL] for *S. aureus* ATCC 25923 (green)and *E. coli* ATCC 259323 (yellow) caused by different coatings; TiAlV used as control.

For *S. aureus*, the initial count of 7.65 × 10^7^ CFU/mL dropped to 2.6 × 10^2^ CFU/mL with the TiAlV/TiN/Chitosan coating, corresponding to a 5.47 log_10_ reduction (99.99% decrease). In the silver-enhanced coating, the count was further reduced to 2.7 × 10^1^ CFU/mL, representing a 6.54 log_10_ reduction (99.99% decrease) (Figure 4).

In the case of *E. coli*, the situation was similar. The initial bacterial count of 7.82 × 10^⁶^ CFU/mL was reduced to 5.0 × 10^3^ CFU/mL with the TiAlV/TiN/Chitosan coating, representing a 3.19 log_10_ reduction (99.93% decrease). When the coating included silver nanoparticles, the count decreased to 4.6 × 10^2^ CFU/mL, corresponding to a 4.13 log_10_ reduction (99.99% decrease) (Figure 2).

The reduction in OD_600_ values was statistically significant for all the tested bacterial strains (ANOVA, *p* < 0.00001). Similarly, the reduction in CFU/mL for *E. coli* and *S. aureus* was significant (*p* < 0.05). The post hoc Tukey’s test confirmed major differences between the control and both coatings in OD_600_ for all tested strains (*p* < 0.01), while CFU/mL values for *E. coli* and *S. aureus* were also visibly reduced (*p* < 0.01) (Appendix A.1.—Statistical Analysis).

According to the CLSI guidelines [26], a reduction of ≥3 log_10_ in bacterial counts is indicative of bactericidal activity. Both examined coatings surpassed this threshold, with the TiAlV/TiN/Chitosan/Ag coating demonstrating superior efficacy across all the tested strains. The results supported the remarkable antibacterial performance of the coatings as they exceeded the established standards for antibacterial efficacy. The best effects were achieved for the TiN/Ag composite. The addition of silver nanoparticles significantly enhanced the antibacterial activity, reducing it up to 6.54 log_10_ (99.99%) of bacterial populations. The statistical analysis confirmed the effectiveness of the TiAlV/TiN/Chitosan and TiAlV/TiN/Chitosan/Ag coatings on bacterial growth inhibition (*p* < 0.01).

### 2.4. Inhibitory Impact of Tested Coatings on Biofilm Formation

**Figure 5:**
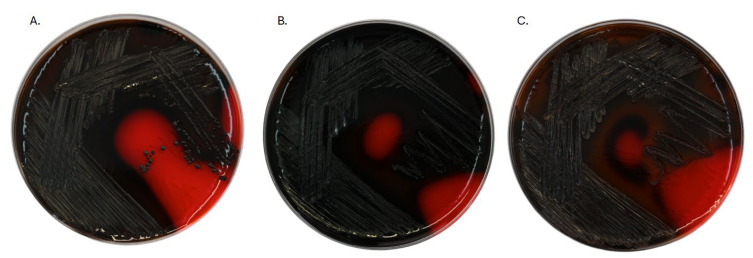
Biofilm formation on Congo Red Agar—qualitative assessment. Positive results (black colony phenotype) for *S. aureus* ATCC 25923 (**A**), *E. faecium* ATCC 700221 (**B**), and *E. faecalis* ATCC 29212 (**C**).

The bacterial ability to produce biofilm was assessed by the Freeman method with Congo Red Agar Plates [27]. Bacterial strains that are capable of producing biofilm would grow on Congo Red Plates as black colonies. In our study, the positive results for biofilm formation were confirmed by the presence of this phenotype. The data regarding *S. aureus* ATCC 25923 (Figure 5A) revealed the colonies creating the typical black colony phenotype, which confirmed the strong biofilm-forming ability of this strain. *S. aureus* is capable of producing an extracellular matrix, which contributes to the persistence of this strain on biomaterial surfaces and its resistance to antimicrobial treatments. *E. faecium* ATCC 700221 (Figure 5B) also displayed the black colony phenotype, indicating its capability to form biofilms. Moreover, *E. faecalis* ATCC 29212 (Figure 5C) also exhibited the black colony phenotype, suggesting its biofilm-forming capacity. This finding highlights the role of *Enterococcus* in biofilm-related infections, particularly in the context of medical implants, where biofilm formation can hinder the proper treatment process.

The Freeman method results confirmed all the tested strains, *S. aureus*, *E. faecium*, and *E. faecalis*, *E. gallinarum* to be capable of producing biofilms. Among them, the *E. gallinarum* strain exhibited the weakest biofilm production.

Such a disadvantageous phenomenon contributes to the persistence and resistance of bacteria in implant-related infections. Therefore, it is of utter importance to develop biomaterials with enhanced antibiofilm properties to prevent risky complications of the treatment.

**Figure 6:**
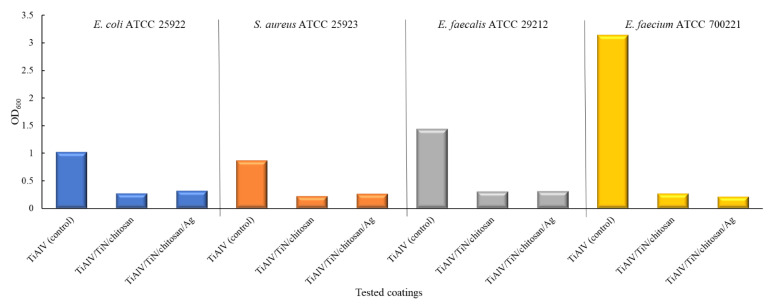
Inhibition of biofilm formation produced by *E. coli* (blue), *S. aureus* (orange), *E. faecalis* (grey), and *E. faecium* (yellow)on tested coatings, expressed as OD_600_ value reductions measured via crystal violet biofilm staining.

The next research step revealed the anti-biofilm potential of the tested coatings. The optical density (OD) of the formed biofilm was reduced in the case of all the bacterial strains. The most significant biofilm inhibition was achieved by the coatings containing silver nanoparticles. The biofilm OD decreased by 69–74% for *E. coli*, for *S. aureus* by 70–75%, for *E. faecium* by 91–93%, and for *E. faecalis* by 78–79%. The use of silver nanoparticles resulted in the biofilm reduction exceeding 68% for all the tested strains, with a particularly notable decrease of over 90% for *E. faecium* (Figure 6, Table 2).

The strain-specific variations emphasize the need for tailored coating strategies with regard to particular pathogens. For instance, silver nanoparticles appear most promising for combating *E. faecium*.

**Figure 7:**
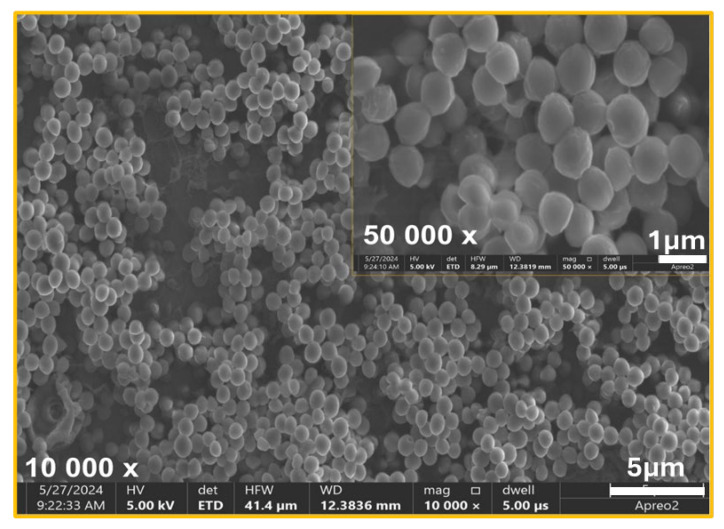
SEM image of bacterial biofilm of *S. aureus* ATCC 25923 on TiAlV (control surface). No apparent disruption or morphological changes visible.

**Figure 8:**
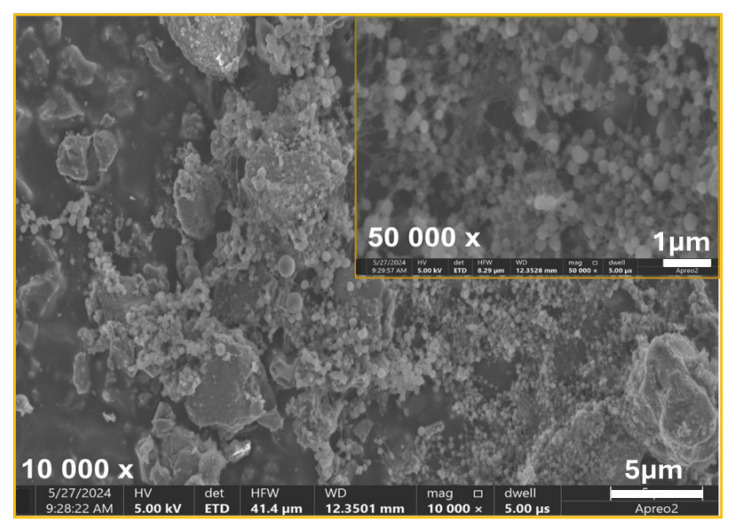
SEM image of bacterial biofilm of *S. aureus* ATCC 25923 on TiAlV/TiN/Chitosan coating. Deformation of bacterial cells and reduced biofilm coverage indicate antibacterial effects of TiAlV/TiN/Chitosan coating.

**Figure 9:**
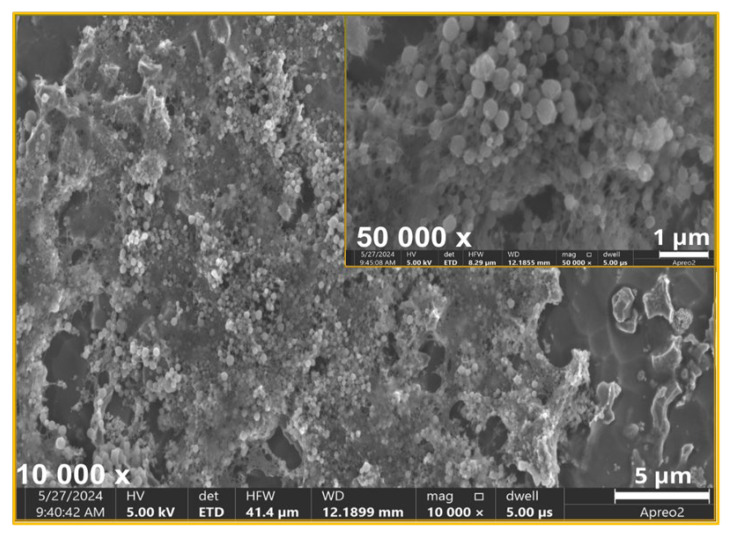
SEM image of bacterial biofilm of *S. aureus* ATCC 25923 on TiAlV/TiN/Chitosan/Ag coating. Antibacterial activity illustrated with noticeable damage to bacterial cells.

Additionally, the SEM analysis was conducted to evaluate the morphology of bacterial cells and biofilm structures. Based on the SEM images, distinct differences in bacterial cell morphology and biofilm structure were observed between the control material (Figure 7) and the TiAlV/TiN/Chitosan (Figure 8). On the control (Figure 7), *S. aureus* cells appeared to maintain their characteristic spherical shape, forming a densely packed biofilm. The biofilm structure was robust and continuous, indicating favorable conditions for bacterial adhesion and colonization. No apparent disruption or morphological changes were visible, suggesting the control material did not inhibit bacterial growth or biofilm formation. While, on the TiAlV/TiN/Chitosan surface (Figure 8), the biofilm was visibly disrupted and less dense. Many bacteria exhibited morphological alterations, e.g., signs of deformation or damage, indicating the antibacterial effects of the TiAlV/TiN/Chitosan coating. The reduced biofilm coverage proved the coating to limit bacterial adhesion and biofilm development, impairing *S. aureus’* ability to form a cohesive biofilm layer. Similar changes in the biofilm morphology and bacterial damage were observed on the material containing silver nanoparticles (TiAlV/TiN/Chitosan/Ag) (Figure 9). On this material, the bacterial count was even lower, highlighting the enhanced antibacterial and antibiofilm properties of the Ag-containing coating.

**Figure 10:**
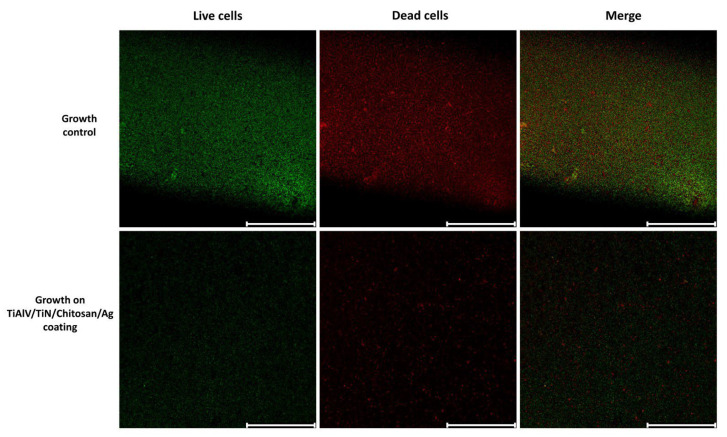
Representative confocal microscopy images showing the viability of *E. faecium* biofilm cells. **Top row**—growth control sample; **bottom row**—biofilm grown on TiAlV/TiN/Chitosan/Ag-coated disks after 18 h incubation. Images display live cells (green, SYTO 9), dead cells (red, PI), and a merged image of both fluorescence signals. Significant reduction in live cells proportion, demonstrating antimicrobial efficacy of chitosan/silver composite coating. Scale bar: 200 µm.

Additionally, to deepen the analysis of the impact of TiAlV/TiN/Chitosan/Ag coating on bacterial cell viability and biofilm structure, we employed confocal microscopy. The SYTO 9/PI fluorescence intensity ratio was used according to the LIVE/DEAD™ BacLight™ Bacterial Viability Kit protocol. For *E. faecium*, the SYTO 9/PI ratio was 0.543 in the growth control sample, indicating a predominance of live cells relative to dead cells. After the 18 h incubation on the tested biomaterial, the ratio decreased to 0.296, reflecting a substantial decline in the biofilm viability. The observed percentage reduction in viability was approximately 45.49%, as compared to the control, indicating significant biofilm damage on the TiAlV/TiN/Chitosan/Ag coating and a marked decrease in the viable cell number after the prolonged exposure (Figure 10). Similarly, for *S. aureus*, the SYTO 9/PI ratio after 18 h was 0.208, i.e., an even more pronounced reduction in the biofilm viability, with a percentage decrease of 63.68% in comparison to the growth control.

## 3. Discussion

The structural differences between Gram-negative and Gram-positive bacteria influence how they respond to antimicrobial compounds of the biofilm-inhibiting coatings, such as chitosan and silver nanoparticles. Gram-negative bacteria (such as *E. coli*) possess a double membrane to endure in varied environments; an additional outer one overlays the thin peptidoglycan layer. The outer membrane contains lipopolysaccharides forming an extremely selective barrier that reduces the permeability of larger molecules, including certain antimicrobial compounds. The double membrane makes Gram-negative bacteria essentially more resistant to external factors like antibiotics and biopolymers. The negatively charged lipopolysaccharides layer may also electrostatically repel cationic antimicrobial compounds (e.g., chitosan) by limiting their ability to bind to and disrupt bacterial membranes. Silver nanoparticles can partially overcome this defense releasing ions that penetrate the bacterial envelope. However, the less successful biofilm inhibition of Gram-negative strains, as compared to Gram-positive ones, suggests that the lipopolysaccharides barrier limits the full impact of both chitosan and silver [28,29,30].

The case of Gram-positive bacteria is different. Strains such as *S. aureus*, *E. faecalis*, and *E. faecium* lack the membrane on the outside and possess a thicker peptidoglycan layer rich in teichoic acids. This layer provides structural strength, which also makes Gram-positive bacteria more prone to antimicrobial resistance. Chitosan has cationic properties and can bind to the peptidoglycan, which disrupts the bacterial cell wall, causing its content leak. Silver ions can penetrate the bacterial structure and interact with components of the cell, which enhances their antimicrobial activities. The superior reduction in the biofilm formation observed in *S. aureus* in comparison to *E. coli* can thus be attributed to these distinctive structural features [28,31].

Our study revealed the essential antibacterial and antibiofilm potential of the coatings based on titanium nitride (TiN). The most promising option is the material doped with silver nanoparticles (AgNPs). The materials were examined with the most common strains in implant-related infections, such as *E. coli*, *S. aureus*, *E. faecalis*, and *E. faecium*. The obtained data underscored the composite coatings as active agents to mitigate the bacterial colonization on biomaterial surfaces. Chitosan displayed efficient antibacterial activity, particularly against *S. aureus*. However, its direct effect was limited concerning *E. coli*. Titanium nitride exhibited strong, broad-spectrum antibacterial properties, making it an excellent base layer for antimicrobial coatings. Its performance in reducing bacterial counts was notable in both tested strains. Silver nanoparticles added to the TN coating amplified the antibacterial effect, nearly eradicating bacterial cells. The mechanism consisted of the Ag’s ability to disrupt bacterial membranes and generate reactive oxygen species.

Other coatings, such as HA-Ag and polymer-based silver composites, also exhibit antibacterial properties, but they have limited durability. In contrast, TiN/Ag stands out due to its high mechanical durability, making it more suitable for long-term implant applications. TiN/Ag effectively reduces the biofilm formation and eliminates bacteria. Its high values of hardness and wear resistance ensure long-term surface protection. While its biocompatibility is promising, long-term silver release requires further in vivo studies. The choice of TiN/Ag is based on its combination of strong antibacterial properties, durability, and potential biocompatibility, making it an attractive option for medical implants [11,32].

AgNPs act against bacteria through several mechanisms. They penetrate membranes, causing structural damage and leakage of cellular contents. They bind to sulfur-containing proteins, leading to membrane rupture and they disrupt metabolic enzymes, causing oxidative stress. Once inside, they generate reactive oxygen species (ROS) that further damage DNA, proteins, and lipids, ultimately leading to bacterial death [33].

The biofilm’s density or composition differs, depending on the bacterial strain, which may influence the efficacy of coatings. *E. faecium* is a Gram-positive species, notorious for forming dense, multilayered biofilms enriched with extracellular polymeric substances, including polysaccharides, proteins, and extracellular DNA. These dense matrices act as physical barriers, reducing the penetration of antimicrobials and shielding embedded bacterial cells from direct exposure. They create microenvironments within the biofilm that can trap nutrients, stabilize pH, and limit the diffusion of reactive oxygen species generated by silver nanoparticles [34,35]. Despite these, in our study, the TiAlV/TiN/Chitosan/Ag coatings significantly (>90%) reduced the biofilm formation for *E. faecium*. Possibly, chitosan disturbed the microbial cell membrane’s integrity, which allowed silver nanoparticles to penetrate the biofilm matrix. Such a phenomenon exerted antimicrobial effects on cells protected within the inner layers [36].

Recent studies suggest that the combination of chitosan and silver nanoparticles can generate ROS which contribute significantly to antimicrobial activity by causing oxidative damage to bacterial cell components, including DNA, proteins, and lipids [37,38,39]. The oxidative stress mechanism complements the physical disruption caused by chitosan and enhances the overall efficacy of these composite coatings. Another study has shown that this ROS-mediated mechanism is essential for the Ag-NPs’ antimicrobial activity, as it disrupts bacterial cell components and biofilms, enhancing their overall efficacy against biofilm-associated infection [40].

In the context of biofilm-associated infections, dense biofilms like those formed by *E. faecium* pose additional challenges. However, the ability of chitosan to disrupt the biofilm matrix, coupled with the penetration of silver ions facilitated by ROS generation, provides a compelling solution for implant-related infections. These findings align with another research that highlights the synergistic potential of chitosan and silver-based strategies in addressing biofilm resilience [38,41,42]. Our results also confirmed the antibacterial activity of these nanoparticles, as the LIVE/DEAD staining revealed a reduction in the bacterial cell viability of approximately 45% and 63% for *E. faecium* and *S. aureus*, respectively. These results support the efficacy of silver nanoparticles in combination with chitosan for biofilm disruption and bacterial inactivation.

The biocompatibility of protective coatings used in medical applications is a crucial factor in implant development. Titanium nitride TiN-based coatings, produced using various available methods, have been extensively studied for their compatibility with biological tissues and their interaction with surrounding cells. Shi Jin et al. [43] investigated the biocompatibility of TiN coatings deposited on a NiTi medical alloy by conducting MTT, adhesion, and apoptosis tests on L-929 murine fibroblast cells. Their studies demonstrated that TiN coatings could enhance cell adhesion, spreading, and proliferation on the NiTi substrate, which indicated improved biocompatibility. Similarly, Xingming Ji et al. [44] examined TiN coatings on a Ti-6Al-4V alloy modified with silver and calcium ions, reporting enhanced osteoblast adhesion and antibacterial properties, further supporting their potential in medical applications.

While TiN coatings are well known for their biocompatibility and mechanical strength, the addition of AgNPs induces their strong antibacterial activity. In that case, the combination of TiN with AgNPs provides a surface that is resistant to microbial colonization but also durable and safe for patients. The pure TiN coating lacked these properties. The incorporation of silver nanoparticles and chitosan into the coating enhanced its antibiofilm and antibacterial characteristics. The obtained results proved our materials’ ability to reduce bacterial cell counts and inhibit biofilm formation. Such an ability might be a great step to develop implants by reducing infection risks. However, a key limitation of this study is the potential long-term cytotoxicity and mechanical wear of TiN and TiN/Ag coatings. Due to friction, Ti-6Al-4V alloys can release toxic vanadium ions, while the prolonged silver release may raise biocompatibility concerns. Although lower silver content in TiN-Ag coatings helps to control release and reduce toxicity, the long-term effects in implant environments require further study [45]. Moreover, while TiN-Ag coatings exhibit antibacterial activity and good adhesion, an increased silver content leads to reduced hardness and wear resistance. This suggests the need for optimizing coating composition to balance antibacterial properties with mechanical performance. Additionally, further in vivo studies are required to comprehensively evaluate the biocompatibility, durability, and effectiveness of these coatings in real biological environments. [46].

## 4. Materials and Methods

### 4.1. Biomaterials

Metallic circular substrates with a diameter of 1 cm were fabricated from grade 5 Ti-6Al-4V alloy and supplied by Wolften (Wrocław, Poland). The tested coatings were applied to the Ti-6Al-4V circle plates using the electrophoretic deposition (EPD) technique.

Titanium nitride particles with an average size smaller than 3 µm, and silver nanoparticles, averaging less than 100 nm, were obtained from Sigma Aldrich, Hamburg, Germany. The EPD suspension was prepared by combining two separately formulated precursor suspensions. The first suspension involved dissolving 0.125 g of chitosan (molecular weight: 100,000–300,000; (Thermo Scientific, Waltham, MA, USA) in 20 mL of distilled water with the addition of 0.9 mL of acetic acid (Pureland, Łódź, Poland). The second suspension was made by dispersing TiN particles in a solvent mixture of 5 mL isopropyl alcohol (Pureland, Poland) and 25 mL ethanol (Poch, Gliwice, Poland). The final suspension contained 0.5 wt% TiN particles.

Each precursor suspension was subjected to 1 h of ultrasonic treatment prior to mixing. Silver nanoparticles (Sigma Aldrich, Hamburg, Germany) were added to half of the combined suspension at a concentration of 1 g/L. Both suspensions were then treated ultrasonically for an additional hour, followed by magnetic stirring for 24 h. Before starting the deposition process, the suspension was sonicated for 2 min to ensure a uniform dispersion.

Before the application of EPD coatings, the titanium alloy plates were cleaned and etched through a multi-step process. Initially, the plates underwent ultrasonic cleaning in acetone (Pureland, Łódź, Poland) for 30 min, followed by another 30 min of ultrasonic cleaning in ethanol (Poch, Poland). Subsequently, the plates were etched in a 5% hydrofluoric acid solution (Chempur, Piekary Śląskie, Poland) for 30 s and rinsed thoroughly with distilled water.

**Figure 11:**
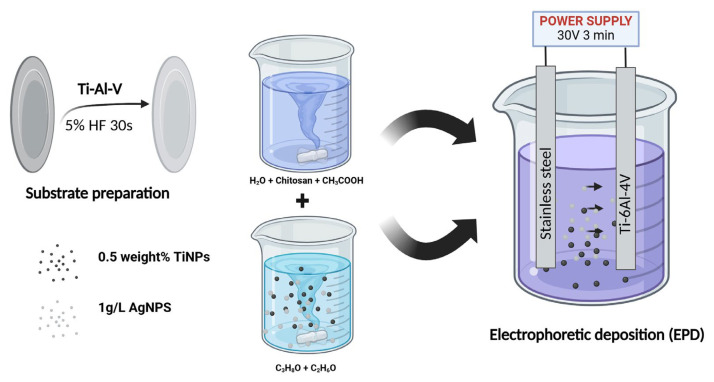
Scheme representing electrophoretic deposition (EPD) of coatings.

The electrophoretic deposition was performed at 30 V for 3 min. The titanium alloy plates acted as the cathode, while a stainless-steel electrode served as the anode. The distance between the electrodes was maintained at 1.5 cm. The sample preparation process is presented in Figure 11.

### 4.2. Antibacterial Activity

#### 4.2.1. Preliminary Qualitative Evaluation of Antibacterial Potential of Solutions

The preliminary assay to evaluate the antibacterial activity of two different solutions containing (1) titanium nitride particles and chitosan (2) titanium nitride particles, chitosan, and silver nanoparticles (Sigma Aldrich, Hamburg, Germany) was conducted against the set of reference strains: *S. aureus* ATCC 25923, *E. coli* ATCC 25922, *E. faecalis* ATCC 29212, *E. faecalis* ATCC 51299, *E. faecium* ATCC 19434, *E. faecium* ATCC 700221, and *E. gallinarum* ATCC 49434. The solutions activity was tested via the disk-diffusion Kirby Bauer method.

Briefly, blank disks (Oxoid, Argenta, Poznań, Poland) were prepared by impregnating each with 20 µL of the tested solutions. Simultaneously, bacterial suspensions were prepared from overnight pure cultures in 0.85% NaCl solution (bioMérieux, Marcy-l’Étoile, France) to achieve turbidity equivalent to 0.5 McFarland units. The suspensions were evenly inoculated onto Mueller–Hinton Agar using a sterile cotton swab, ensuring full coverage. After the inoculation, the disks impregnated with the tested compounds were placed onto the agar surface. The plates were incubated aerobically at 37 °C for 24 h. Following the incubation, the diameters of complete growth inhibition zones were measured in millimeters.

A growth inhibition zone diameter of ≥10 mm was used as the breakpoint to classify bacteria as susceptible to the tested solution [47]. The cutoff was established based on the evaluation of the solvent’s impact on the inhibition zone, which did not exceed 10 mm.

#### 4.2.2. Quantitative Evaluation of Antibacterial Activity

The quantitative antibacterial activity against *S. aureus* ATCC 25923, *E. coli* ATCC 25922, *E. faecalis* ATCC 29212, and *E. faecium* ATCC 700221 of selected biomaterials was evaluated according to the slightly modified ASTM E 2180-07 norm [48,49,50].

The bacterial preparation process began with an overnight culture of the bacteria in 50 mL of TSB broth (Tryptic-soy broth, BD BBL™, Franklin Lakes, NJ, USA). Then, a bacterial suspension was created from each pure culture and inoculated into an agar mixture (which was composed of 0.7% Brain Heart Infusion (BHI) agar, Oxoid, Basingstoke, UK, and 0.85% NaCl) to achieve a final concentration of 105 CFU/mL (colony-forming units per mL). A 50 µL aliquot of the inoculated agar was subsequently applied onto the tested biomaterials placed in a 24-well flat-bottom plate (NEST Biotechnology, Cambridge, MA, USA). After the agar gelled, the samples were incubated at 37 °C for 24 h. In this procedure, bacteria were cultured overnight in 50 mL of Tryptic Soy Broth (TSB, BD BBL™, Franklin Lakes, NJ, USA). From the cultures, bacterial suspensions of each strain were created and introduced into an agar mixture composed of 0.7% Brain Heart Infusion (BHI) agar (Oxoid, UK) and 0.85% NaCl, resulting in a final concentration of 5 × 105 colony-forming units per milliliter (CFU/mL). A 50 µL aliquot of the slurry was placed onto the materials in 24-well flat-bottom plates (NEST biotechnology) and left to solidify. The samples were incubated at 37 °C for 24 h. After incubation, each sample was carefully transferred to a fresh tube containing 2 mL of Brain Heart Infusion (BHI) broth (Oxoid, UK) to release and collect any adhered bacteria. To facilitate the bacteria detachment from the biomaterial surfaces, an ultrasonic bath treatment was applied for 1 min, followed by an additional 1 min vortex mixing at room temperature. This process ensured the maximum dislodgement of bacterial cells from the material surfaces into the broth. Following the detachment, serial dilutions of the bacterial suspension were prepared in BHI broth within a series of 2 mL Eppendorf tubes. The diluting process began with a 10-1 dilution and proceeded through six steps, ending with a 10-6 dilution, each carefully mixed to ensure consistent bacterial distribution across the tubes. From each diluted sample, a 100 µL aliquot was then spread onto Mueller Hinton Agar (Oxoid, UK) plates to allow for accurate colony formation and bacterial quantification. The agar plates were subsequently incubated at 37 °C for a 24 h period, allowing for visible colony development. Colony counts from the highest dilution exhibiting bacterial growth were used to calculate the concentration of viable bacteria originally present on the biomaterial surface, ensuring that only live, culturable bacteria were included in the final quantification.

Finally, the number of bacterial colonies on the plate showing the highest dilution with observable growth was counted. The concentration of surviving bacteria on the tested biomaterials was calculated using the following formula:

CFU/mL = X × 0.1 × R

where X stands for the count of bacterial colonies observed on the plate at the highest dilution level that still shows detectable growth. The term R represents the corresponding dilution factor for that plate, ranging from 10^−1^ to 10^−6^ depending on which dilution level contained the viable colonies. Each experiment was conducted in triplicate to ensure accuracy.

### 4.3. Antibiofilm Activity

Firstly, we selected the strains based on a phenotypic evaluation of their biofilm production capability. Using Congo Red, we assessed which bacteria produced the most biofilm. Bacteria from pure cultures were inoculated onto CRA (Congo Red Agar, London UK) plates (Congo Red, Sigma Aldrich, and Brain Heart Infusion BHI agar, Oxoid). The plates were then incubated for 24 h, and then we observed changes in the color of the medium. According to the literature [27,51], after incubation, the black color indicated a high capacity for extracellular slime production, characteristic of biofilm formation. The brown color suggested a moderate capacity, while no color change signified the absence of extracellular slime production and, thus, the inability to form biofilms.

Next, the antibiofilm activity was evaluated using the Christensen method [52], which involves staining the biofilm with crystal violet. The procedure began with cultivating microorganisms on appropriate media that allowed the biofilm formation on special disks. After incubation, the plates were carefully washed to remove unattached cells. The biofilm was then fixed using 99% ethanol, and after the evaporation of any residual alcohol, the biofilm was stained with crystal violet for a specified duration. The excess stain was removed by washing the plates with distilled water, and the plates were left to dry. The concentration of the bound dye reflected the actual biofilm amount and was measured spectrophotometrically at a wavelength of 600 nm (OD_600_).

### 4.4. SEM Analysis

The layer’s coatings microstructure, the morphology evaluation of bacteria, and the biofilm formation on the investigated samples were performed using the Apreo 2S low vac high-resolution scanning electron microscope from Thermo Fisher Scientific. Prior to imaging, all the samples were coated with a thin conducting layer of carbon (5 nm) and observed with an ETD detector at an accelerating voltage of 5 kV. The elemental content of the active modifiers incorporated in the layers was examined using the EDAX Octane Elite energy dispersive x-ray spectroscopy (EDS/EDX) system with Octane Elite silicon drift detectors (SDDs) operated with APEX™ Advanced 2022 software, version 2.5.1001.001. The qualitative analysis was performed using a standardless method.

### 4.5. Bacterial Cell Viability Assay and Confocal Microscopy Analysis

The antibacterial activity of the surface and the biofilm cell viability were assessed by means of the LIVE/DEAD™ BacLight™ Bacterial Viability Kit (Thermo Fisher, Waltham, MA, USA). The assay was performed according to the manufacturer’s instructions.

Briefly, the bacterial biofilm was cultured on the surface of the tested biomaterial disks, specifically TiAlV/TiN/Chitosan/Ag, identified before as having the highest antibacterial activity. After the 18 h incubation, the samples were washed twice with PBS and subsequently dried. The samples were then stained using a prepared solution containing propidium iodide (PI) and SYTO 9 in a 1:1 ratio, diluted 10×, and then fixed by mounting oil (Thermo Fisher, Waltham, MA, USA). SYTO 9 stained live cells by emitting fluorescence in the range of 509–529 nm, while PI (propidium iodide) stained dead cells by emitting fluorescence in the range of 609–629 nm. The calculated SYTO 9/PI ratio represented the relative proportion of live to dead cells in the sample. According to the manufacturer’s guidelines, the fluorescence intensity ratio was expected to show a linear relationship with the percentage of live cells in the sample. The biofilm samples cultured on biomaterial disks were stained with a solution containing SYTO 9 and PI in a 1:1 ratio, diluted 10-fold [53].

Images were obtained using a Leica Stellaris 8 WLL DLS confocal microscope (Leica, Wetzlar, Germany). Each scan was performed using the same microscope settings, including resolution (2048 × 2048 pixels), laser power and frequency, detector power, pinhole (1.0 AU), and z-stack (approximately 14 focal planes). The samples were scanned with a 20× objective lens (NA 0.4). The obtained images were analyzed using LAS X software (version 4.5) (Leica, Germany) to evaluate the mean fluorescence intensity. The results were expressed as the ratio of green to red fluorescence intensity (live to dead cells), according to the cell viability assay employed in the study. The scalebar represented 200 µm.

Each measurement was run in six replicates to ensure the reliability and accuracy of the results.

### 4.6. Statistical Analysis

The data were analyzed using the one-way analysis of variance (ANOVA) to determine significant differences in the bacterial growth inhibition determined by OD_600_ and CFU/mL (*p* < 0.05 for significant results). Tukey’s HSD post hoc test was performed for multiple comparisons. The statistical analysis was conducted using Statistica (version 13.0).

## 5. Conclusions

In conclusion, the tested coatings demonstrated significant antibacterial and antibiofilm activity against all the studied strains, showcasing their potential for use as implantable biomaterials. The titanium nitride coatings, particularly those enhanced with silver nanoparticles, exhibited promising potential in reducing the bacterial biofilm formed on the tested surfaces. Such an innovative solution not only offers robust antibacterial and antibiofilm properties but also ensures mechanical durability. Therefore, the developed coatings appear a significant advancement in the field of biomaterials for medical implants as they mitigate risks of implant-associated infections.

However, further research is necessary to validate the coating’s efficacy in clinical settings, optimize its application processes, and assess long-term biocompatibility to ensure safe and effective integration into medical practice. Additionally, future studies should focus on in vivo evaluations to investigate their clinical applicability, long-term stability, and overall safety in real-world medical scenarios. While the findings demonstrate the high potential of titanium nitride coatings with silver nanoparticles, certain limitations should be acknowledged. Scaling up the production of such coatings while maintaining their uniformity and effectiveness presents a challenge. Additionally, the long-term antimicrobial efficacy of these coatings in dynamic biological environments requires further investigation to ensure their sustained performance. Addressing these aspects in future studies will be crucial for their successful clinical translation.

## Figures and Tables

**Table 1:** Inhibition zone diameter [mm] for tested bacterial strains after treatment with TiN/Chitosan and TiN/Chitosan/Ag.

Bacterial Strains	Tested Solutions	
	TiN/Chitosan [mm]	TiN/Chitosan/Ag [mm]
*E. coli* ATCC 25922 [19]	12	18
*S. aureus* ATCC 25923 [20]	14	20
*E. faecalis* ATCC 29212 [21]	15	17
*E. faecalis* ATCC 51299 [22]	14	16
*E. faecium* ATCC 19434 [23]	15	16
*E. faecium* ATCC 700221 [24]	16	22
*E. gallinarum* ATCC 49434 [25]	14	15

**Table 2:** Reduction in biofilm formation produced by *E. coli*, *S. aureus*, *E. faecalis*, and *E. faecium* on different types of coatings.

Bacterial Strain	Type of Coating	% of Biofilm Reduction
*E. coli* 25922	TiAlV/TiN/Chitosan	74%
	TiAlV/TiN/Chitosan/Ag	69%
*S. aureus* 25923	TiAlV/TiN/Chitosan	75%
	TiAlV/TiN/Chitosan/Ag	70%
*E. faecalis* 29212	TiAlV/TiN/Chitosan	79%
	TiAlV/TiN/Chitosan/Ag	78%
*E. faecium* 700221	TiAlV/TiN/Chitosan	91%
	TiAlV/TiN/Chitosan/Ag	93%

**Figure A1:**
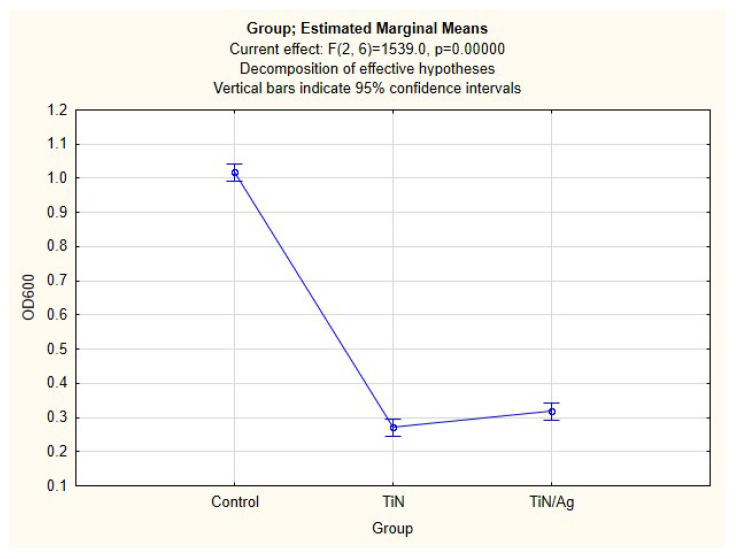
One-way ANOVA results showing reduction in bacterial growth (OD600) in *E. coli* 25922 on different types of coatings. Statistically significant differences observed among tested groups (*p* < 0.05).

**Figure A2:**
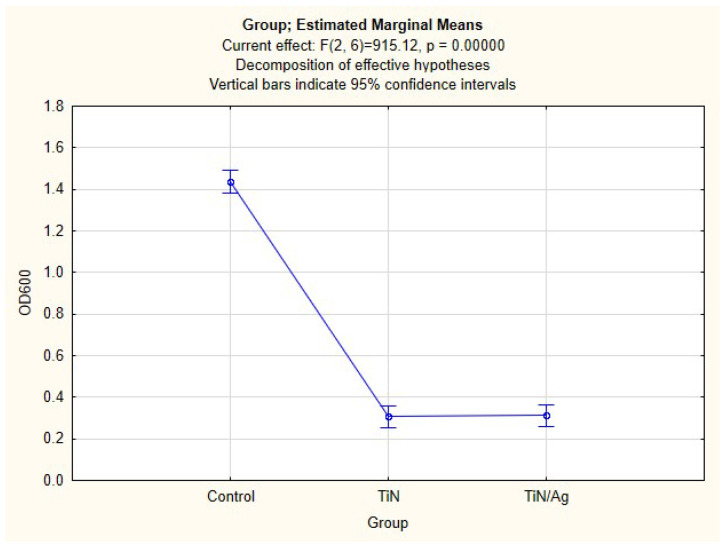
One-way ANOVA results showing reduction in bacterial growth (OD600) in *E. faecalis* 29212 on different types of coatings. Statistically significant differences observed among tested groups (*p* < 0.05).

**Figure A3:**
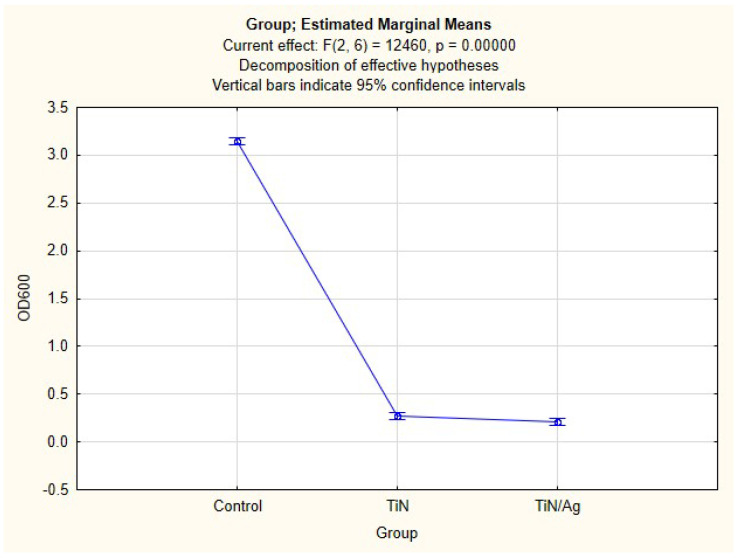
One-way ANOVA results showing reduction in bacterial growth (OD600) in *E. faecium* 700221 on different types of coatings. Statistically significant differences observed among tested groups (*p* < 0.05).

**Figure A4:**
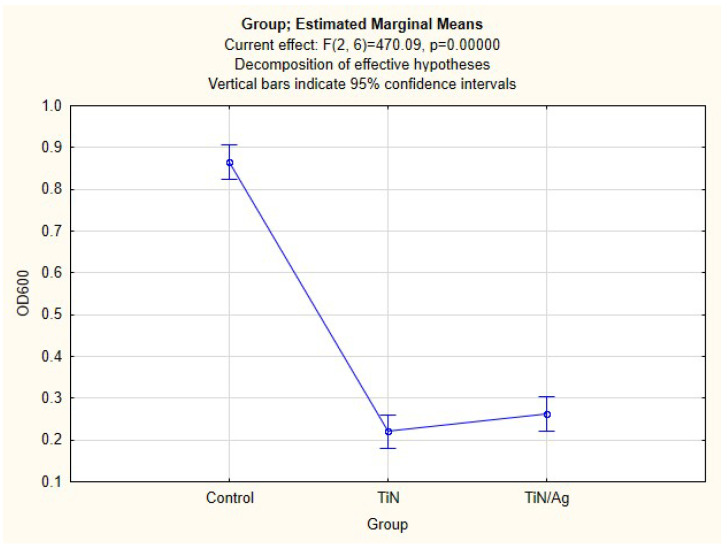
One-way ANOVA results showing reduction in bacterial growth (OD600) in *S. aureus* ATCC 25923 on different types of coatings. Statistically significant differences observed among tested groups (*p* < 0.05).

**Figure A5:**
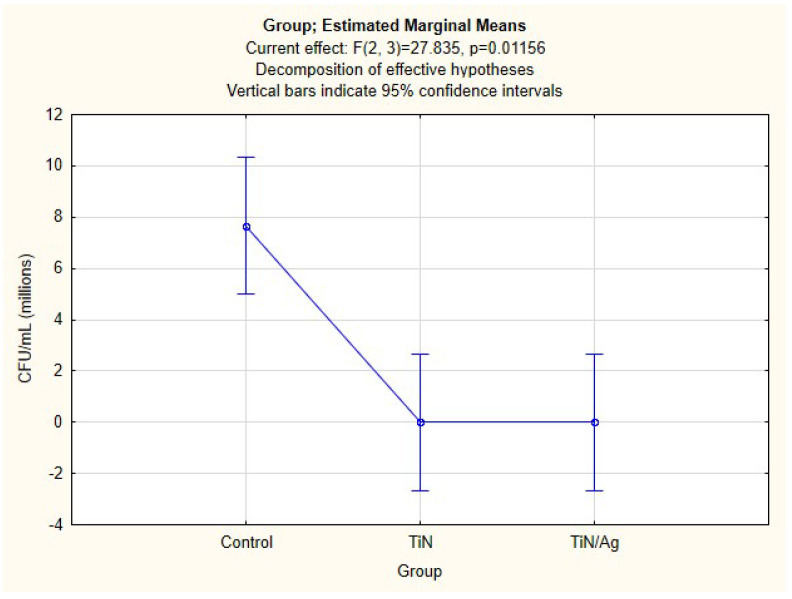
One-way ANOVA results illustrating reduction in viable bacterial cells (CFU/mL) for *S. aureus* ATCC 25923 on different coatings. Analysis revealed significant differences between tested groups (*p* < 0.05).

**Figure A6:**
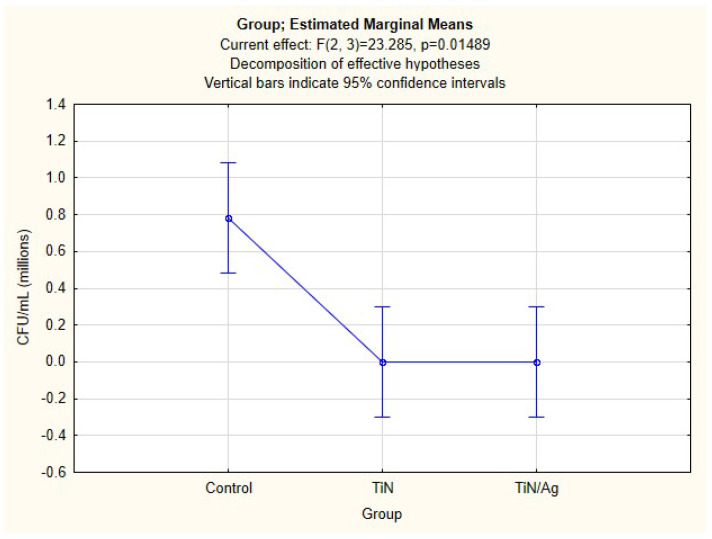
One-way ANOVA results illustrating reduction in viable bacterial cells (CFU/mL) for *E. coli* 25922 on different coatings. Analysis revealed significant differences between tested groups (*p* < 0.05).

## Data Availability

The original contributions presented in this study are included in the article. Further inquiries can be directed to the corresponding authors.

## Abbreviations

The following abbreviations are used in this manuscript:

AgNPs	silver nanoparticles
ATCC	American-type culture collection
CFU	colony forming units
EPD	electrophoretic deposition
HF	hydrofluoric acid
PI	propidium iodine
TiN	titanium nitride
SEM	scanning electron microscopy

## Appendix A

### Appendix A.1. Statistical Analysis

**Table A1:** Results of ANOVA analysis for reduced bacterial growth (OD600 and CFU/mL) in *E. coli*, *S. aureus*, *E. faecalis*, and *E. faecium* on different types of coatings.

Strain	Group	OD600	*p* (OD600)	CFU/mL	*p* (CFU/mL)
*E. coli* 25922	Control	High	*p* < 0.00001	High	0.01489
	TiAlV/TiN/Chitosan	Significantly reduced		Significantly reduced	
	TiAlV/TiN/Chitosan/Ag	Significantly reduced		Significantly reduced	
*S. aureus* 25923	Control	High	*p* < 0.00001	High	0.01156
	TiAlV/TiN/Chitosan	Significantly reduced		Significantly reduced	
	TiAlV/TiN/Chitosan/Ag	Significantly reduced		Significantly reduced	
*E. faecium* 700221	Control	High	*p* < 0.00001	Not tested	
	TiAlV/TiN/Chitosan	Significantly reduced			
	TiAlV/TiN/Chitosan/Ag	Significantly reduced			
*E. faecalis* 29212	Control	High	*p* < 0.00001		
	TiAlV/TiN/Chitosan	Significantly reduced			
	TiAlV/TiN/Chitosan/Ag	Significantly reduced			

**Table A2:** Results of Tukey’s HSD post hoc test showing pairwise comparisons of bacterial growth reduction (OD600 and CFU/mL) between control and tested coatings for *E. coli*, *S. aureus*, *E. faecalis*, and *E. faecium*.

Strain	Reference	Treatment	*p* (OD600)	*p* (CFU/mL)
*E. coli* 25922	Control	TiAlV/TiN/Chitosan	0.000227	0.019777
		TiAlV/TiN/Chitosan/Ag		
*S. aureus* 25923	Control	TiAlV/TiN/Chitosan	0.000227	0.015424
		TiAlV/TiN/Chitosan/Ag		
*E. faecium* 700221	Control	TiAlV/TiN/Chitosan	0.000227	
		TiAlV/TiN/Chitosan/Ag		
*E. faecalis* 29212	Control	TiAlV/TiN/Chitosan	0.000227	
		TiAlV/TiN/Chitosan/Ag		
